# Diferenças entre homens e mulheres na prevalência da fragilidade e fatores associados entre adultos mais velhos: evidências do ELSI-Brasil 

**DOI:** 10.1590/0102-311XPT144923

**Published:** 2024-04-22

**Authors:** Silvia Lanziotti Azevedo da Silva, Geraldo Eduardo Guedes de Brito, Nair Tavares Milhem Ygnatios, Juliana Vaz de Melo Mambrini, Maria Fernanda Lima-Costa, Juliana Lustosa Torres

**Affiliations:** 1 Universidade Federal de Juiz de Fora, Juiz de Fora, Brasil.; 2 Universidade Federal da Paraíba, João Pessoa, Brasil.; 3 Núcleo de Estudos em Saúde Pública e Envelhecimento, Fundação Oswaldo Cruz/Universidade Federal de Minas Gerais, Belo Horizonte, Brasil.; 4 Instituto René Rachou, Fundação Oswaldo Cruz, Belo Horizonte, Brasil.; 5 Programa de Pós-graduação em Saúde Pública, Universidade Federal de Minas Gerais, Belo Horizonte, Brasil.; 6 Universidade Federal de Minas Gerais, Belo Horizonte, Brasil.

**Keywords:** Fragilidade, Saúde do Idoso, Exercício Físico, Frailty, Health of the Elderly, Exercise, Fragilidad, Salud del Anciano, Ejercicio Físico

## Abstract

Este trabalho, baseado em amostra nacional representativa da população com 50 anos ou mais, objetivou estimar a prevalência da fragilidade entre homens e mulheres, identificar fatores sociodemográficos e de saúde associados e estimar a fração atribuível populacional. Foram utilizados dados da segunda onda (2019-2021) do *Estudo Longitudinal da Saúde dos Idosos Brasileiros* (ELSI-Brasil). A fragilidade foi classificada pelo número de itens positivos entre perda de peso não intencional, exaustão, baixo nível de atividade física, lentidão da marcha e fraqueza. As análises principais foram baseadas na regressão logística multinomial estratificada por sexo. A prevalência da fragilidade foi menor nos homens (8,6%; IC95%: 6,9; 10,7) do que nas mulheres (11,9%; IC95%: 9,6; 14,8), sendo o item mais frequente o baixo nível de atividade física em ambos. A idade e a escolaridade foram os fatores sociodemográficos associados à pré-fragilidade e à fragilidade entre homens e mulheres. Houve diferença da fração atribuível populacional para fragilidade entre os sexos. Nos homens, a maior fração atribuível populacional foi para não ter companheiro (23,5%; IC95%: 7,7; 39,2) e escolaridade baixa (18,2%; IC95%: 6,6; 29,7). Nas mulheres, maiores frações atribuíveis populacionais foram para déficit de memória (17,1%; IC95%: 7,6; 26,6), déficit da visão (13,4%; IC95%: 5,1; 21,7) e diabetes mellitus (11,4%; IC95%: 4,6; 18,1). Observou-se fração atribuível populacional semelhante para doença cardíaca (8,9%; IC95%: 3,8; 14,1, em mulheres; e 8,8%; IC95%: 2,0; 15,6, em homens). Estratégias voltadas para a prática de atividade física têm o potencial de prevenir a fragilidade em ambos os sexos, enquanto a prevenção de condições crônicas é mais importante nas mulheres.

## Abstract (en)

Based on a national representative sample of the population aged 50 years or older, this study aimed to estimate the prevalence of frailty among men and women, identify associated sociodemographic and health factors, and estimate the population attributable fraction. Data from the second wave (2019-2021) of the *Brazilian Longitudinal Study of Aging* (ELSI-Brazil) were used. Frailty was classified based on the number of positive items among unintentional weight loss, exhaustion, low level of physical activity, slow gait, and weakness. The main analyses were based on multinomial logistic regression stratified by sex. The prevalence of frailty was lower in men (8.6%; 95%CI: 6.9; 10.7) than in women (11.9%; 95%CI: 9.6; 14.8), with the most frequent item being the low level of physical activity in both. Age and schooling level were the sociodemographic factors associated with pre-frailty and fragility among men and women. The population attributable fraction was different for frailty between genders. In men, the highest population attributable fraction was due to not having a partner (23.5%; 95%CI: 7.7; 39.2) and low schooling level (18.2%; 95%CI: 6.6; 29,7). In women, higher population attributable fraction values were due to memory deficit (17.1%; 95%CI: 7.6; 26.6), vision deficit (13.4%; 95%CI: 5.1; 21.7), and diabetes mellitus (11.4%; 95%CI: 4.6; 18,1). Similar population attributable fraction levels were observed for heart disease (8.9%; 95%CI: 3.8; 14.1 in women and 8.8%; 95%CI: 2.0; 15.6 in men). Strategies aimed at physical activity have the potential to prevent frailty in both men and women, and the prevention of chronic conditions is more important in women.

## Abstract (es)

Este estudio tuvo por objetivo estimar, utilizando una muestra nacional representativa de la población de 50 años o más, la prevalencia de la fragilidad entre hombres y mujeres, identificar los factores sociodemográficos y de salud asociados, y calcular la fracción atribuible a la población. Se utilizaron datos de la 2ª ola (2019-2021) del *Estudio Longitudinal de Salud de los Ancianos Brasileños* (ELSI-Brasil). La fragilidad se clasificó por el número de elementos positivos entre pérdida de peso no intencional, agotamiento, bajo nivel de actividad física, marcha lenta y debilidad. Los principales análisis se basaron en la regresión logística multinomial estratificada por sexo. La prevalencia de fragilidad fue menor en los hombres (8,6%; IC95%: 6,9; 10,7) que en las mujeres (11,9%; IC95%: 9,6; 14,8), y como ítem más frecuente el bajo nivel de actividad física en ambos. La edad y el nivel educativo fueron los factores sociodemográficos asociados a la pre-fragilidad y la fragilidad entre hombres y mujeres. Hubo una diferencia en fracción atribuible a la población para la fragilidad entre los sexos. Entre los hombres, la fracción atribuible a la población más elevada fue no tener pareja (23,5%; IC95%: 7,7; 39,2) y bajo nivel educativo (18,2%; IC95%: 6,6; 29,7). Entre las mujeres, las fracción atribuible a la población más elevadas fueron déficit de memoria (17,1%; IC95%: 7,6; 26,6), déficit de visión (13,4%; IC95%: 5,1; 21,7) y diabetes mellitus (11,4%; IC95%: 4,6; 18,1). Se observaron fracción atribuible a la población similares para enfermedades cardíacas (8,9%; IC95%: 3,8; 14,1 en mujeres, y 8,8%; IC95%: 2,0; 15,6 en hombres). Las estrategias dirigidas a la actividad física tienen el potencial de prevenir la fragilidad en ambos sexos, mientras que la prevención de enfermedades crónicas es más necesaria en las mujeres.

## Introdução

A fragilidade é considerada uma síndrome referente a um estado de vulnerabilidade e à perda da capacidade adaptativa das pessoas mais velhas, perante as demandas físicas e ambientais, podendo levar a desfechos negativos, como incapacidade e morte ^1^. Uma revisão da literatura localizou 67 instrumentos para avaliar a fragilidade na pesquisa e prática clínica, sendo o fenótipo proposto por Fried et al. ^2^ para a fragilidade física o mais usado em estudos com diversos objetivos como avaliação etiológica, estimativa de prevalência e risco de fragilidade, estudos metodológicos, biomarcadores, diretrizes e desfecho de intervenções ^3^. O fenótipo de fragilidade é baseado em cinco itens, incluindo perda de peso não intencional, exaustão, baixo nível de atividade física, lentidão da marcha e fraqueza, classificando indivíduos como frágeis (≥ 3 itens positivos), pré-frágeis (1-2 itens positivos) ou não frágeis (nenhum item positivo) ^2^.

Em relação à prevalência da fragilidade física, a literatura aponta uma variação entre 4% e 17%, de acordo com fatores como localização geográfica, média de idade dos participantes do estudo e residência ou não na comunidade ^4^. No Brasil, segundo uma metanálise ^5^ baseada em 18 estudos, a prevalência da fragilidade física foi de 16% (intervalo de 95% de confiança - IC95%: 13,0; 19,0) em pessoas com 60 anos ou mais. Apesar da fragilidade ser mais estudada na população idosa, dados da primeira onda do *Estudo Longitudinal da Saúde dos Idosos Brasileiros* (ELSI-Brasil), conduzida em 2015-2016, com amostra nacional representativa da população com 50 anos ou mais, identificou 9,1% de participantes frágeis ^6^. Mais ainda, identificou que desses, 58% apresentaram a concomitância de fragilidade e presença de multimorbidade ^6^, demostrando que ações preventivas para evitar essa síndrome, que vem aumentando em gerações mais recentes ^7^, já podem ser iniciadas em idades mais jovens, por meio de ações gerais de promoção da saúde e prevenção de agravos.

Fatores de risco para isso são estabelecidos na literatura ^8^. Entre os fatores sociodemográficos, destaca-se a idade avançada ^9^^,^^10^^,^^11^ e a baixa escolaridade ^9^^,^^11^. Entre os fatores relacionados a condições de saúde, destacam-se a presença de doenças cardiovasculares ^9^ e diabetes mellitus ^11^, a multimorbidade ^9^^,^^12^ e o déficit cognitivo ^9^^,^^11^^,^^12^. Entretanto, as revisões e metanálises citadas ^9^^,^^10^^,^^11^ não distinguem a estratificação por sexo.

Em relação ao local do estudo, grande parte foi conduzido em países de renda alta, como países da Europa. No Brasil, a maioria dos estudos foi conduzido na Região Sudeste ^5^ ou restritos a cidades específicas, como Santa Cruz (Rio Grande do Norte), Campinas (São Paulo), São Paulo e Belo Horizonte (Minas Gerais). Com representatividade nacional, foi encontrado apenas um estudo, que avaliou fatores associados à fragilidade, sendo identificado somente a baixa escolaridade como fator associado ^13^. Assim, é importante estabelecer melhor os fatores associados em amostras de representatividade nacional que possam apresentar relevância para elaboração e implementação de estratégias preventivas populacionais, no âmbito nacional.

Portanto, o objetivo deste estudo foi estimar a prevalência da fragilidade entre homens e mulheres com 50 anos ou mais, residentes no Brasil, identificar fatores sociodemográficos e de saúde associados à fragilidade, assim como estimar a fração atribuível populacional para a fragilidade nessa população.

## Métodos

### Fonte de dados

Trata-se de um estudo transversal utilizando dados da segunda onda (conduzida em 2019-2021) do ELSI-Brasil, um estudo longitudinal representativo da população brasileira com 50 anos ou mais. Os procedimentos para a seleção da amostra baseiam-se em métodos probabilísticos, que combinam estratificação em três estágios de seleção (município, setor censitário e domicílio), abrangendo 70 municípios das cinco grandes regiões do Brasil ^14^. A cada onda subsequente, há reposição da amostra para garantir a representatividade nacional da amostra ^15^. A amostra da segunda onda incluiu 9.949 participantes. Informações mais detalhadas do ELSI-Brasil podem ser vistas em publicações anteriores ^14^^,^^15^.

O ELSI-Brasil foi aprovado pelo Comitê de Ética da Fundação Oswaldo Cruz (Fiocruz), Minas Gerais (protocolo nº 34649814.3.0000.5091). Todos os participantes assinaram o Termo de Consentimento Livre e Esclarecido (TCLE).

### Fragilidade

A fragilidade (não frágil, pré-frágil ou frágil) foi definida de acordo com o número de itens positivos, segundo os critérios estabelecidos por Fried et al. ^2^, incluindo perda de peso não intencional, exaustão, baixo nível de atividade física, lentidão da marcha e fraqueza. A categoria não frágil incluiu aqueles indivíduos que não apresentaram nenhum item positivo; pré-frágil incluiu aqueles com um ou dois itens positivos; e a categoria frágil incluiu aqueles que apresentaram três ou mais itens positivos. Cada um dos itens positivos foi avaliado conforme descrito a seguir:

(1) Perda de peso não intencional: autorrelato de perda de peso não intencional maior do que 4,5kg nos últimos três meses;

(2) Exaustão: frequência de três dias ou mais na semana para pelo menos uma das duas perguntas sobre sintomas depressivos, obtidos por meio da escala do *Center for Epidemiological Studies - Depression* (CES-D), previamente validada para a população brasileira ^16^, como descrito a seguir: “Na última semana, com que frequência o(a) Sr(a). sentiu que não conseguiria levar adiante suas coisas (iniciava alguma coisa, mas não conseguia terminar)?”; “Na última semana, com que frequência a realização de suas atividades rotineiras exigiram do(a) Sr(a). um grande esforço para serem realizadas?”;

(3) Baixo nível de atividade física: foi definida pelo quintil inferior de gasto calórico semanal, em quilocalorias, estratificado segundo sexo. Para o cálculo do gasto calórico semanal, foi utilizada a versão reduzida do *International Physical Activity Questionnaire* (IPAQ), também previamente validada para a população brasileira ^17^, que avalia atividades físicas leves, moderadas e vigorosas realizadas na última semana, por pelo menos dez minutos continuamente. O ponto de corte do quintil inferior foi semelhante para homens e mulheres (zero quilocaloria);

(4) Lentidão da marcha: foi definida pelo quintil superior de tempo gasto para percorrer três metros, em segundos, estratificado segundo sexo e altura (altura média de 166cm para homens e 155cm para mulheres). Para a mensuração do tempo gasto para percorrer três metros, foi utilizado um cronômetro, considerando-se o menor tempo em duas tentativas. Aqueles indivíduos impossibilitados de realizar o teste foram incluídos no quintil superior. Detalhamento dessa medida pode ser consultado em publicação prévia ^18^. O ponto de corte do quintil superior dos homens foi de 11,6 segundos para alturas menores ou iguais à média e de 15,3 segundos para alturas acima da média. Já para as mulheres, o ponto de corte do quintil superior foi de 10,5 segundos para alturas menores ou iguais à média e de 10,0 segundos para alturas acima da média.

(5) Fraqueza: foi definida pelo quintil inferior de força de preensão palmar, em quilogramas, estratificada segundo sexo e quartis de índice de massa corporal - IMC (mensurado objetivamente dividindo-se o peso em quilogramas pela altura ao quadrado em metros - kg/m^2^). A mensuração da força de preensão palmar no membro dominante foi realizada por um dinamômetro manual, considerando-se a maior força registrada entre três tentativas. Aqueles indivíduos impossibilitados de realizar o teste foram considerados no quintil inferior. Maiores detalhes dessa medida foram publicados anteriormente ^19^. O ponto de corte do quintil inferior dos homens variou de 21kg para IMC menor que 24,5kg/m^2^ até 27kg/m^2^ para IMC maior ou igual a 30,4kg/m^2^. Quartis intermediários de IMC tiveram ponto de corte de 24kg (IMC entre 24,5 e 27,2kg/m^2^) e de 25kg (IMC entre 27,3 e 30,3kg/m^2^). Já para as mulheres, o ponto de corte do quintil inferior variou de 13kg para IMC menor que 25,4kg/m^2^ até 15kg para IMC maior ou igual a 32,9kg/m^2^. Quartis intermediários de IMC tiveram ponto de corte de 14kg (IMC entre 25,4 e 28,7kg/m^2^) e de 15kg (IMC entre 28,8 e 32,8kg/m^2^).

### Potenciais fatores associados à fragilidade

Os potenciais fatores associados incluíram variáveis sociodemográficas e de saúde. As variáveis sociodemográficas consideradas foram: idade (50-59, 60-69, ou ≥ 70 anos); escolaridade (0-1, 2-8 ou ≥ 9 anos completos de estudo); e estado conjugal (com ou sem companheiro(a)). As variáveis de saúde incluíram o autorrelato de diagnóstico médico para diabetes mellitus (não ou sim); doença cardíaca, incluindo infarto, angina ou insuficiência cardíaca (não ou sim); e hipertensão arterial (não ou sim). Além dessas, foram consideradas a percepção de déficit de visão (não ou sim) e audição (não ou sim), classificando-se como “déficit” quando o participante avaliou a sua visão (de perto ou de longe) ou sua audição como ruim/muito ruim. Por último, incluiu-se a função cognitiva, avaliada por meio do teste de memória de aprendizagem de lista de dez palavras, somando-se o número de palavras repetidas logo após a leitura das palavras (memória imediata) e o número de palavras repetidas após cinco minutos de sua leitura inicial (memória tardia), variando de 0 a 20 palavras ^20^. De acordo com o resultado dos testes, definiu-se a variável déficit de memória (não ou sim), sendo o “déficit” classificado pelo ponto de corte de um desvio padrão abaixo da média, ou seja, valores abaixo de cinco palavras ^20^.

### Análise estatística

Primeiramente, foi verificada a distribuição das características sociodemográficas e de saúde e seus respectivos IC95%, para o conjunto de participantes e segundo o sexo. Foi utilizado o teste qui-quadrado de Pearson com correção de Rao Scott para a verificação de diferenças entre os sexos, considerando-se um nível de significância de 5%. Posteriormente, foram estimadas as prevalências das categorias de fragilidade e seus IC95%, assim como as prevalências de seus itens positivos, para todos os participantes e segundo o sexo. Para avaliar a associação entre características sociodemográficas e de saúde e a classificação da fragilidade, foram utilizados os modelos de regressão logística mutinomial com estimativas de *odds ratio* (OR) e seus IC95%, adotando-se como referência a categoria “não frágil” e ajustados por todas as características simultaneamente. Baseado nos modelos ajustados, foram estimadas a fração atribuível populacional ^21^ da fragilidade (≥ 3 itens positivos), que estima em que proporção essa condição seria prevenida na população caso as características modificáveis estatisticamente associadas (p < 0,05) à fragilidade fossem eliminadas. Essa estimativa foi feita separadamente para cada sexo. A fração atribuível populacional combina a força de associação da variável verificada no modelo de regressão e a prevalência dessa variável na população. Todas as análises foram realizadas no software Stata/SE, versão 17.0 (https://www.stata.com), considerando-se o desenho do estudo e o peso amostral dos indivíduos.

## Resultados

Dos 9.949 participantes da segunda onda do ELSI-Brasil, 6.424 tinham informações completas para a classificação da fragilidade e foram incluídos nesta análise, totalizando 2.508 homens e 3.916 mulheres. A média de idade dos homens foi de 63,8 anos (desvio padrão - DP = 8,8 anos) e das mulheres, 63,3 anos (DP = 9,6 anos). A Tabela 1 mostra a distribuição das características sociodemográficas e de saúde dos participantes, para o conjunto de adultos mais velhos e segundo o sexo. Homens e mulheres diferiram estatisticamente em relação à presença de companheiro(a), diagnóstico de diabetes mellitus e hipertensão arterial e percepção de déficit de visão. Em relação aos homens, as mulheres apresentaram maior prevalência de diabetes mellitus (19,4% *vs*. 14,6%), de hipertensão arterial (53% *vs*. 42,1%) e de déficit de visão (22,3% *vs*. 19,4%) e menor proporção de viver com um(a) companheiro(a) (52,5% *vs*.72,7%).

**Tabela 1:** Distribuição das características sociodemográficas e de saúde dos participantes, entre total de participantes e segundo sexo. *Estudo Longitudinal da Saúde dos Idosos Brasileiros* (ELSI-Brasil), 2019-2021.

Variáveis	Total (n = 6.424) *		Homens (n = 2.508) *		Mulheres (n = 3.916) *		Valor de p **
	%	IC95%	%	IC95%	%	IC95%	
Variáveis sociodemográficas							
Idade (anos)							0,564
50-59	45,5	42,0; 49,1	44,8	40,9; 48,7	46,1	42,2; 50,1	
60-69	30,2	28,7; 31,7	30,1	28,1; 32,2	30,3	28,3; 32,4	
≥ 70	24,3	21,3; 27,5	25,1	21,2; 29,6	23,6	20,9; 26,5	
Escolaridade (anos completos de estudo)							0,901
0-1	20,8	17,2; 25,1	20,7	17,1; 24,8	21,1	16,4; 26,6	
2-8	51,3	48,5; 54,2	51,1	47,7; 55,6	51,6	48,2; 54,9	
≥ 9	27,8	24,4; 31,6	28,2	24,3; 32,4	27,3	23,4; 31,7	
Estado conjugal							< 0,001
Com companheiro(a)	61,4	57,9; 64,8	72,7	69,6; 75,7	52,5	48,7; 56,3	
Variáveis de saúde							
Diabetes mellitus	17,3	15,4; 19,3	14,6	12,4; 17,0	19,4	16,9; 22,2	0,004
Doença cardíaca	7,6	6,7; 8,8	8,0	6,7; 9,5	7,4	6,2; 8,8	0,451
Hipertensão arterial	48,2	45,2; 51,2	42,1	38,7; 45,5	53,0	49,7; 56,2	< 0,001
Percepção de déficit de visão	21,0	17,8; 24,6	19,4	15,9; 23,5	22,3	19,0; 26,0	0,031
Percepção de déficit de audição	5,7	4,6; 6,69	5,8	4,5; 7,5	5,6	4,3; 7,1	0,779
Déficit de memória ***	20,9	18,1; 23,9	21,1	17,6; 25,1	20,7	17,8; 23,9	0,809

IC95%: intervalo de 95% de confiança.

* Valores sem considerar os parâmetros amostrais;

** Baseado no teste qui-quadrado de Pearson com correção de Rao-Scott;

*** Avaliada pelo teste de memória imediata e tardia de aprendizagem de lista de palavras.

Em relação à fragilidade e aos itens utilizados para a sua classificação, a Tabela 2 mostra que os itens da fragilidade positivos seguem o mesmo padrão entre homens e mulheres, sendo que o item mais frequente para a classificação da fragilidade foi o baixo nível de atividade física, relatado por 31,1% dos homens e 36,1% das mulheres, seguido do item fraqueza, em 26,7% dos homens e 27,8% das mulheres. Os itens que foram estatisticamente maiores nas mulheres em comparação aos homens foram a exaustão e a baixa atividade física. Quando agrupados pelo baixo nível de fragilidade, percebe-se que as mulheres apresentaram uma prevalência de fragilidade (11,9%) maior que os homens (8,6%), totalizando uma prevalência de fragilidade de 10,5% (IC95%: 8,6; 12,7).

**Tabela 2:** Prevalência de cada um dos itens positivos utilizados para a classificação fragilidade e a prevalência da fragilidade, entre total de participantes e segundo sexo. *Estudo Longitudinal da Saúde dos Idosos Brasileiros* (ELSI-Brasil), 2019-2021.

	Total (n = 6.424) *		Homens (n = 2.508) *		Mulheres (n = 3.916) *		Valor de p **
	%	IC95%	%	IC95%	%	IC95%	
Itens da fragilidade positivos							
Perda de peso não intencional (> 4,5kg nos últimos três meses)	4,6	3,7; 5,7	4,1	3,1; 5,5	4,9	3,9; 6,1	0,207
Exaustão (frequência de sintomas em três ou mais dias da semana em uma de duas perguntas da CES-D)	18,4	15,4; 22,0	15,0	11,9; 18,7	21,2	17,9; 24,9	< 0,001
Baixo nível de atividade física (quintil inferior do gasto calórico semanal pelo IPAQ)	33,9	29,2; 38,9	31,1	26,2; 36,5	36,1	30,9; 41,7	0,026
Lentidão da marcha no quintil inferior	18,0	13,3; 24,0	18,6	13,0; 25,8	17,6	13,2; 23,0	0,547
Fraqueza (quintil inferior da força da preensão palmar)	27,3	23,9; 30,9	26,7	22,1; 31,8	27,8	24,5; 31,3	0,610
Classificação da fragilidade							0,047
Não frágil (nenhum item positivo)	34,8	30,1; 39,9	36,6	30,0; 43,8	33,5	29,5; 37,7	
Pré-frágil (1-2 itens positivos)	54,7	50,7; 58,7	54,8	49,0; 60,4	54,6	50,8; 58,4	
Frágil (≥ 3 itens positivos)	10,5	8,6; 12,7	8,6	6,9; 10,7	11,9	9,6; 14,8	

CES-D: *Center for Epidemiological Studies - Depression Scale*; IC95%: intervalo de 95% de confiança; IPAQ: *International Physical Activity Questionnaire*.

* Valores sem considerar os parâmetros amostrais;

** Baseado no teste qui-quadrado de Pearson com correção de Rao-Scott.

A Tabela 3 mostra os resultados das análises multivariadas da associação entre características sociodemográficas e de saúde e classificação da fragilidade, segundo o sexo. Os fatores sociodemográficos consistentemente associados à pré-fragilidade e à fragilidade em homens e mulheres foram a idade e a escolaridade, de modo que a chance de fragilidade foi maior nas idades de 70 anos ou mais e menor em escolaridades de nove anos ou mais de estudo. A presença de companheiro(a) associou-se negativamente à fragilidade apenas entre os homens (OR = 0,47; IC95%: 0,27; 0,79). Em relação às características de saúde, percebe-se que as mulheres apresentaram mais características associadas à fragilidade do que os homens. Entre os homens, a fragilidade associou-se positivamente ao diagnóstico de doença cardíaca (OR = 2,25; IC95%: 1,09; 4,66) e déficit de memória (OR = 1,92; IC95%: 1,06; 3,48). Por outro lado, entre as mulheres, além da fragilidade ter sido associada positivamente ao diagnóstico de doença cardíaca (OR = 3,79; IC95%: 2,19; 6,54) e déficit de memória (OR = 3,18; IC95%: 2,05; 4,95), ela também foi positivamente associada ao diagnóstico de diabetes mellitus (OR = 2,02; IC95%: 1,36; 2,98), à percepção de déficit de visão (OR = 2,80; IC95%: 1,80; 4,36) e à percepção de déficit de audição (OR = 2,42; IC95%: 1,26; 4,65). Nenhuma característica de saúde associou-se à pré-fragilidade entre os homens. Já nas mulheres, essa condição associou-se ao diagnóstico de doença cardíaca (OR = 1,76; IC95%: 1,17; 2,66), à percepção de déficit de visão (OR = 1,65; IC95%: 1,24; 2,20) e ao déficit de memória (OR = 1,79; IC95%: 1,22; 2,63).

**Tabela 3:** Resultados das análises de regressão logística multinomial múltipla dos fatores associados à pré-fragilidade (1-2 itens positivos) e à fragilidade (≥ 3 itens positivos) em homens e mulheres. *Estudo Longitudinal da Saúde dos Idosos Brasileiros* (ELSI-Brasil), 2019-2021.

Variáveis	Homens (n = 1.936)				Mulheres (n = 3.089)			
	Pré-fragilidade		Fragilidade		Pré-fragilidade		Fragilidade	
	OR	IC95%	OR	IC95%	OR	IC95%	OR	IC95%
Idade (*vs*. 50-59 anos)								
60-69	1,08	0,83; 1,41	1,12	0,64; 1,97	1,20	0,90; 1,59	1,53	0,93; 2,45
≥ 70	1,85	1,33; 2,57	2,37	1,21; 4,67	1,81	1,30; 2,53	5,67	3,87; 8,03
Escolaridade (*vs*. 0-1 ano completo de estudo)								
2-8	0,84	0,61; 1,16	0,44	0,25; 0,79	0,60	0,43; 0,82	0,51	0,34; 0,77
≥ 9	0,63	0,41; 0,98	0,25	0,10; 0,63	0,44	0,30; 0,65	0,42	0,23; 0,78
Estado conjugal (*vs*. sem companheiro(a))	0,83	0,60; 1,14	0,47	0,27; 0,79	1,08	0,87; 1,33	1,10	0,82; 1,49
Diabetes mellitus (*vs*. não)	1,04	0,69; 1,55	1,35	0,69; 2,66	1,15	0,84; 1,58	2,02	1,36; 2,98
Doença cardíaca (*vs*. não)	0,86	0,55; 1,35	2,25	1,09; 4,66	1,76	1,17; 2,66	3,79	2,19; 6,54
Hipertensão arterial (*vs*. não)	1,19	0,92; 1,54	0,78	0,48; 1,29	1,06	0,81; 1,39	1,22	0,81; 1,81
Percepção de déficit de visão (*vs*. não)	1,08	0,77; 1,51	1,74	0,95; 3,17	1,65	1,24; 2,20	2,80	1,80; 4,36
Percepção de déficit de audição (*vs*. não)	1,81	0,91; 3,58	2,20	0,94; 5,14	1,31	0,79; 2,16	2,42	1,26; 4,65
Déficit de memória (*vs*. não)	1,33	0,92; 1,94	1,92	1,06; 3,48	1,79	1,22; 2,63	3,18	2,05; 4,95

IC95%: intervalo de 95% de confiança; OR: *odds ratio*.

Nota: categoria de referência: não frágeis.

**Figura 1:**
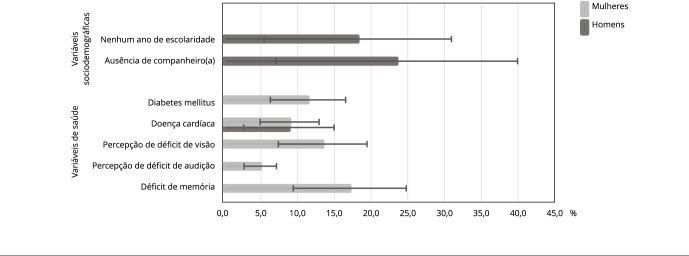
Fração atribuível populacional ajustada das características sociodemográficas e de saúde para a fragilidade (≥ 3 itens positivos), segundo o sexo. *Estudo Longitudinal da Saúde dos Idosos Brasileiros* (ELSI-Brasil), 2019-2021.

A Figura 1 mostra as estimativas da fração atribuível populacional ajustadas da fragilidade (≥ 3 itens positivos), para homens e mulheres, segundo as características sociodemográficas e de saúde que foram independentemente associadas à fragilidade na análise de regressão logística multinomial múltipla. Entre os homens, a maior fração atribuível populacional para a fragilidade foi para a ausência de um companheiro (23,5%; IC95%: 7,7; 39,2), seguida pela escolaridade de até um ano completo (18,2%; IC95%: 6,6; 29,7) e diagnóstico médico para doença cardíaca (8,8%; IC95%: 2,0; 15,6). Entre as mulheres, as maiores frações atribuíveis populacionais foram para déficit de memória (17,1%; IC95%: 7,6; 26,6), percepção de déficit da visão (13,4%; IC95%: 5,1; 21,7), diagnóstico médico para diabetes mellitus (11,4%; IC95%: 4,6; 18,1), diagnóstico médico para doença cardíaca (8,9%; IC95%: 3,8; 14,1) e percepção de déficit de audição (4,9%; IC95%: 0,1; 9,8). Em ambos os sexos, as frações atribuíveis populacionais foram semelhantes apenas para diagnóstico médico para doença cardíaca.

Nota: variáveis testadas, mas não significativas: déficit de memória (homens), escolaridade (mulheres); fração atribuível ajustadas por idade, hipertensão arterial e demais variáveis listadas na tabela. Categoria de referência: não frágeis (nenhum item positivo).

## Discussão

Este estudo, baseado em amostra nacional representativa da população brasileira com 50 anos ou mais, evidenciou uma prevalência da fragilidade semelhante entre homens e mulheres, sendo, no geral, de 10,5%. O item da fragilidade mais prevalente na população estudada foi o baixo nível de atividade física (cerca de um terço da amostra estudada). Em ambos os sexos, a idade mais alta e a baixa escolaridade apresentaram associações independentes com a pré-fragilidade e a fragilidade. Aqueles com doenças do coração e déficit de memória foram mais propensos a apresentar fragilidade, tanto entre homens quanto entre as mulheres. A percepção do déficit de visão apresentou associação com a fragilidade entre as mulheres, mas não entre os homens.

Os fatores sociodemográficos associados à fragilidade (idade e escolaridade) entre homens e mulheres corroboram achados de revisões sistemáticas publicadas anteriormente com estudos sem a estratificação por sexo ^9^^,^^11^. A fragilidade pode gerar declínios cumulativos nos sistemas fisiológicos que aumentam a vulnerabilidade homeostática e desencadeiam alterações no estado de saúde ^22^^,^^23^.

A escolaridade é um importante determinante social da saúde e uma revisão sistemática da literatura internacional aponta relação inversa entre o nível de escolaridade e a progressão da fragilidade ^11^. Especificamente no Brasil, pessoas idosas com maior escolaridade vivem menos anos com fragilidade ^24^ e pessoas frágeis com escolaridade de até oito anos completos apresentam uma propensão 77% maior de hospitalização quando comparada a pessoas frágeis com escolaridade acima de oito anos completos ^25^. Apesar da idade não ser passível de intervenção, o aumento da escolaridade nas novas gerações tem grande potencial de diminuir a prevalência da fragilidade a longo prazo.

A prevalência da fragilidade encontrada neste estudo (10,5%) foi semelhante à observada em 2015-2016 entre os participantes do ELSI-Brasil (9,1%) ^6^, demostrando tendência de estabilização. Entretanto, o aumento foi maior no sexo feminino entre as ondas (de 9,5% ^6^ para 11,9%) em relação ao sexo masculino (de 8,5% ^6^ para 8,6%), provavelmente pela maior expectativa de vida no sexo feminino ^7^. O item do fenótipo da fragilidade que apresentou maior prevalência entre homens e mulheres foi o baixo nível de atividade física, que, apesar de ser medido pelo quintil inferior, apresentou um aumento geral de 19,8% para 33,9%, quando comparado a dados de 2015-2016 na população do ELSI-Brasil ^6^.

O baixo nível de atividade física encontrado é ainda mais preocupante, dado que 57,8% dos brasileiros mais velhos com esse baixo nível passam, também, pelo menos 165 minutos por dia na posição sentada ^26^, caracterizando um comportamento sedentário associado. Deve-se salientar que evidências apontam a relação entre o baixo nível de atividade física, fraqueza e lentidão da marcha em pessoas mais velhas ^27^^,^^28^, de modo que a força muscular adequada é fundamental para preservar a mobilidade funcional e evitar a sarcopenia ^29^. Portanto, reforça-se o fortalecimento de programas voltados à prática de atividade física, como o programa Academia da Saúde, que incluam diversos tipos de atividade física associados à resistência ^30^ para a prevenção e/ou tratamento da fragilidade em pessoas mais velhas.

Com referência à fração atribuível populacional, importantes diferenças foram encontradas entre homens e mulheres. Para os homens, a presença de um(a) companheiro(a) apresentou a maior fração atribuível para a fragilidade, o que pode ser explicado pelo fato de o apoio social dos homens ser muito focado em seu(sua) companheiro(a), enquanto as mulheres fazem maiores trocas com filhos(as) ou outros confidentes ^31^^,^^32^. Tais resultados foram observados na França ^31^ e Inglaterra ^32^, corroborando a tendência internacional da importância do apoio social.

Segundo a força tarefa da Conferência Internacional de Pesquisa sobre Fragilidade e Sarcopenia (2019) ^30^, é fortemente recomendado o provimento de apoio social às pessoas frágeis para que elas sigam corretamente as recomendações do plano de cuidado, principalmente para aquelas em maior vulnerabilidade. O apoio social é passível de intervenção pública, por meio de programas, como o programa Maior Cuidado, desenvolvido em Belo Horizonte, que promove apoio social instrumental a pessoas idosas com maior vulnerabilidade ^33^. Contudo, promover a presença de um cônjuge é mais uma questão cultural. Esses achados, juntamente com a associação encontrada entre fragilidade e escolaridade, evidenciam que a fragilidade física reflete um construto multidimensional e é impactada por questões sociais e familiares ^11^^,^^34^.

Por outro lado, para as mulheres, as maiores frações atribuíveis populacionais foram observadas para condições crônicas, em consonância a resultados de revisões sistemáticas prévias sobre a associação entre fragilidade e diabetes mellitus ^11^ e doenças cardiovasculares ^9^, independentemente do sexo. Para as mulheres, o diabetes mellitus foi associado à fragilidade entre inglesas de 37 a 73 anos ^35^. As mulheres apresentam uma maior prevalência de condições crônicas e fragilidade ^36^, relacionada, também, à maior procura por serviços de saúde ^37^ e acesso ao diagnóstico. Apesar disso, elas ainda apresentam mais barreiras para o tratamento continuado do diabetes mellitus, incluindo as socioculturais e econômicas ^38^, do que os homens, o que decorre dos níveis de escolaridade mais baixos na população feminina ^39^.

Além disso, sabe-se que as condições cardiovasculares, quando bem controladas na atenção primária à saúde, podem prevenir hospitalizações por essas condições ^40^, conhecidas como condições sensíveis à atenção primária. Para além do controle de condições crônicas, a atenção primária, quando bem desenvolvida, tem o potencial de atenuar hospitalizações em pessoas frágeis ^41^. Desse modo, destaca-se o fortalecimento da Política Nacional de Atenção Básica, que embasa os cuidados em saúde ofertados pelo Sistema Único de Saúde (SUS).

Mesmo que este estudo tenha encontrado que a ausência de déficit de memória possa resultar em menor prevalência da fragilidade somente entre as mulheres, uma revisão de estudos longitudinais aponta que o déficit cognitivo é um dos fatores que desencadeiam piores trajetórias da fragilidade ao longo do tempo ^11^. Evidências de uma metanálise mostraram melhora da função cognitiva de pessoas mais velhas sem déficit cognitivo ou com déficits leves por meio da prática atividade física ^42^, que não requer tecnologias avançadas em saúde e pode ser facilmente implantadas na atenção primária à saúde em grupos ou na atenção individualizada. Cabe ressaltar que o rastreio de déficit de cognitivo, juntamente ao déficit visual e auditivo, foram recentemente incorporados pela Organização Mundial da Saúde (OMS) ^43^ nos itens que devem ser rastreados na atenção primária pela Atenção Integrada para os Idosos (ICOPE, do inglês: *Integrated Care for Older People*), independentemente do sexo.

Este estudo apresenta vantagens e limitações. A principal vantagem é a representatividade nacional dos resultados, seguida de ser o primeiro estudo de base nacional examinando os fatores associados à fragilidade separadamente entre homens e mulheres. Entre as limitações do estudo, pode-se destacar a perda amostral de 35%, principalmente devido ao caráter objetivo de dois itens que compõem o fenótipo da fragilidade (lentidão da marcha e fraqueza), dada principalmente pelo não consentimento a essas medidas. Isso sugere que as prevalências encontradas podem estar subestimadas, já que a recusa da realização de medidas físicas pode estar associada à sua pior performance ^44^. De qualquer forma, a prevalência da fragilidade encontrada nesta análise (8,6% nos homens e 11,9% nas mulheres) foi semelhante à observada em estudos brasileiros anteriores ^5^^,^^6^. Outra limitação refere-se ao desenho do estudo transversal, que limita a direcionalidade das associações encontradas.

Sendo a fragilidade uma condição reversível, os resultados reforçam a importância de estratégias populacionais, sobretudo o incremento da atividade física entre adultos mais velhos brasileiros, já que o baixo nível de atividade física foi o item mais frequente para a classificação da fragilidade entre homens e mulheres. A baixa escolaridade apresentou associações consistentes com a fragilidade em ambos os sexos, indicando que o aumento da escolaridade na população tem grande potencial para a redução da prevalência da fragilidade, particularmente nas atuais e futuras coortes mais jovens. Especificamente em relação aos indicadores das condições de saúde, o diabetes mellitus, as doenças cardiovasculares e a qualidade da visão e/ou audição aparecem como fatores independentemente associados à fragilidade entre homens e/ou entre mulheres. Estratégias populacionais ou individuais para a redução dessas condições crônicas na população têm o potencial de reduzir a carga da fragilidade de adultos mais velhos brasileiros.
